# An energy-efficient failure detector for vehicular cloud computing

**DOI:** 10.1371/journal.pone.0191577

**Published:** 2018-01-19

**Authors:** Jiaxi Liu, Zhibo Wu, Jian Dong, Jin Wu, Dongxin Wen

**Affiliations:** School of Computer Science and Technology, Harbin Institute of Technology, Harbin, China; Beijing University of Posts and Telecommunications, CHINA

## Abstract

Failure detectors are one of the fundamental components for maintaining the high availability of vehicular cloud computing. In vehicular cloud computing, lots of RSUs are deployed along the road to improve the connectivity. Many of them are equipped with solar battery due to the unavailability or excess expense of wired electrical power. So it is important to reduce the battery consumption of RSU. However, the existing failure detection algorithms are not designed to save battery consumption RSU. To solve this problem, a new energy-efficient failure detector 2E-FD has been proposed specifically for vehicular cloud computing. 2E-FD does not only provide acceptable failure detection service, but also saves the battery consumption of RSU. Through the comparative experiments, the results show that our failure detector has better performance in terms of speed, accuracy and battery consumption.

## Introduction

Vehicular cloud computing is a promising paradigm that aims at merging mobile cloud computing and vehicular networking, in order to give arise to integrated communication-computing platforms [1]. In vehicular cloud computing, vehicles can be either the service providers to enrich existing cloud services by providing various on-road information (e.g., traffic condition like Pics-on-Wheels proposed by [2]) or be the service consumers to enjoy existing centralized Internet cloud services [3–5]. One of the key features of vehicular cloud computing is high mobility [5–9]. The running job may be interrupted by the random arrival and departure of vehicles [3,8]. We must be able to address the mobility and provide an effective control scheme to guide service conditions and cloud resources [10–11]. Thus fault-tolerant schemes are designed to provide reliable and continuous services in vehicular cloud computing despite the unavailable of some vehicles [12–17]. As an essential building block of the fault-tolerant scheme, a failure detector (FD) plays a critical role in the engineering of such dependable systems [18–19]. An optimized FD should find the vehicles’ failure in a timely and accurate manner [20].

To improve connectivity of vehicular cloud computing, the Roadside Unit (RSU) is deployed along the road [21–23]. For example, many future Internet applications [24] which are delay and delay-jitter sensitive (such as Netflix and VTube) are benefit from RSU. Normally, they use the solar as power input due to the unavailability or excess expense of wired electrical power [25–28]. The solar power are easily affected by the natural environment, e.g., solar power cannot be acquired at night or cloudy day. The energy supply of RSU is unstable, so the energy capacity of RSU is limited. According to the U.S. Department of Transportation [29], it is estimated that 40% of all initial rural freeway roadside infrastructure would have to be solar powered by 2050. A breakdown of the deployment costs also found that over 63% of these roadside infrastructure costs would be consumed by solar energy provisioning, e.g., solar panels, batteries, and their associated electronics. Thus, it is important to reduce the energy consumption of RSUs [28,30].

Existing main adaptive FDs (such as Chen FD [31], *φ*-FD [32], ED FD [33] etc.) keep a sliding window (*WS*) that contains information about received messages to make an estimate of the state (trusted or suspected of having failed) of a monitored node. These FDs need a certain memory space to save a large history message window. At each detection cycle, a large amount of calculation is needed to compute the probability distribution parameters and detector parameters [34]. For most RSUs with battery, these overhead of FDs can exacerbate the battery consumption.

In this paper, aiming at the RSUs with battery, we have presented the Energy-Efficient Failure Detector (2E-FD). It does not rely on the probability distribution of message transmission delay, or on the maintenance of history message windows. We use the arrival time of last message to estimate the arrival time of next message. In addition, the dynamic safety margin, which is computed by the single exponential smoothing method, is used to improve the accuracy of failure detection. To evaluate the performance of 2E-FD, some best-known existing FDs are selected in terms of detection time, mistake rate and query accuracy probability. Besides, we also measure the battery consumption of RSU with different FDs. The experimental results show that 2E-FD is able to reduce the battery consumption of RSU and provide an adaptive failure detection service with high accuracy.

The rest of this paper is organized as follows. In section 2, the related work of vehicular cloud computing and failure detectors is introduced. Section 3 introduces the system model and presents the implementation of 2E-FD. Section 4 carries out the experiments on real traces and tests the battery consumption of RSU with different FDs. Finally, the work is concluded in section 5.

## Related work

In this section, vehicular cloud computing is firstly introduced. Second, the quality of service (QoS) metrics of FD is introduced. Finally, several existing main adaptive FDs are presented.

### Vehicular cloud computing

**Fig 1:**
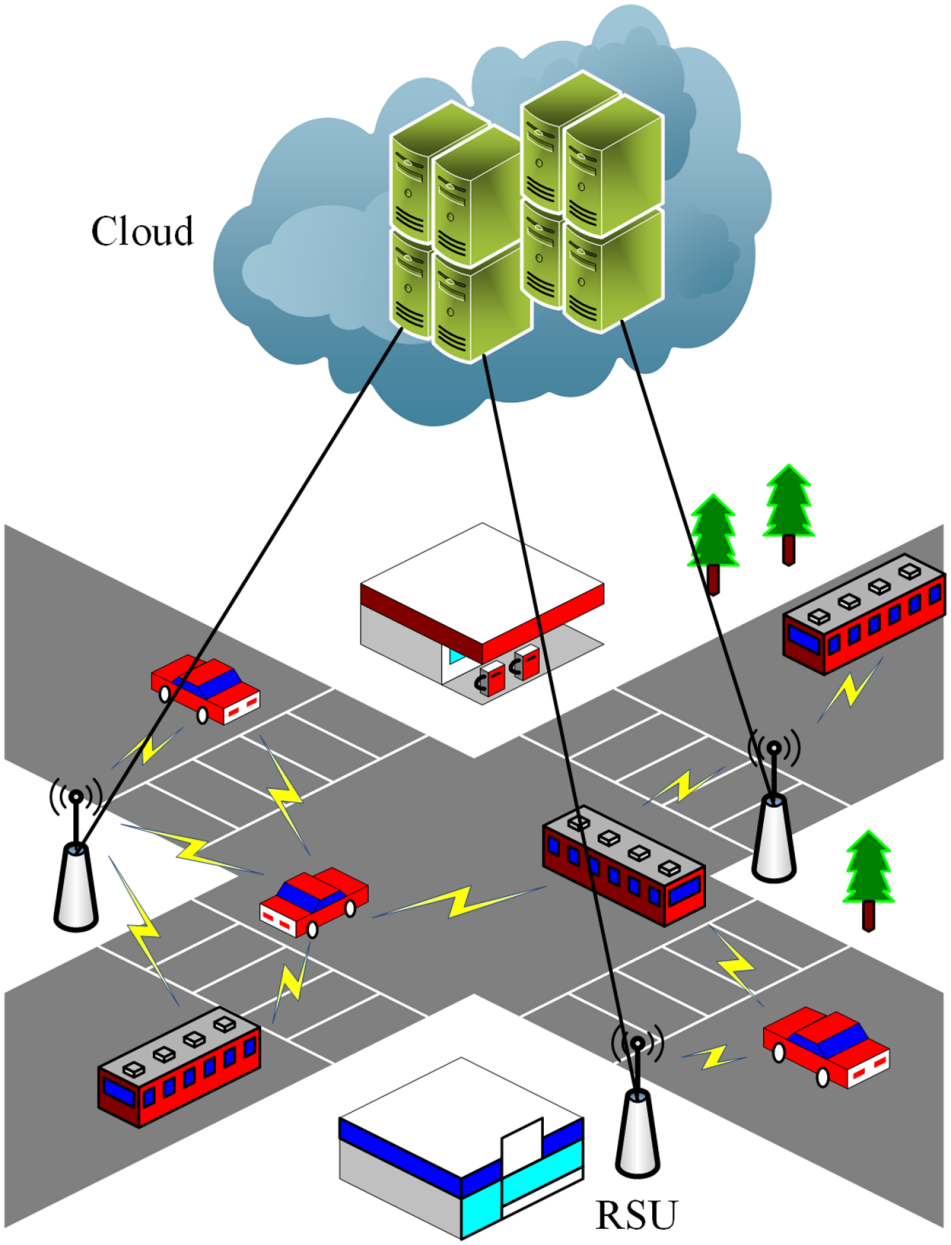
Architecture of vehicular cloud computing.

Olariu et al. [35–37] advocate the concept of vehicular cloud, which coordinates the computing, sensing, communication and storage resources to provide services to authorized users. Different from conventional Internet cloud with dedicatedly installed hardware, vehicular cloud leverages the already available resources on vehicles. In vehicular cloud computing, vehicles communicate with the data centers where the wanted cloud services locate using vehicles-to-infrastructure (V2I) communications, e.g., LTE, WiMax. In addition, vehicles can also work in an autonomous way solely relying on the vehicle-to-vehicle (V2V) communication capabilities. Accordingly, we think that vehicular cloud can be described by a loose two-tier architecture. The first-tier is the Internet cloud computing platform (e.g., data centers) while the second-tier consists of many vehicular cloudlet [3]. The vehicular cloudlet is made up of RSU and vehicles. A user can acquire cloud services from either the first-tier data center or the second-tier vehicular cloudlet. An architecture example is illustrated in Fig 1.

### QoS metrics of failure detector

Many distributed applications have some timing constraint on the behaviors of FDs [19,38]. It is not acceptable that a node is suspected hours after than it has crashed or the FD outputs several false positives. To solve this problem, Chen [31] proposed a series of metrics to specify the QoS of FD: how fast it detects actual failures and how well it avoids false detections. These metrics can quantitatively represent the detection speed and accuracy. We use *T* or *S* to represent whether a node is trusted or suspected. *T*-transition means that the output of the detector changes from *S* to *T*, while *S*-transition means that the output of the detector changes from *T* to *S*. The following three primary metrics are used to describe the QoS of a FD.

Detection time (*T*_*D*_) is the time that elapses from the moment when a node crashes to the time when it starts being suspected, i.e., when the final *S*-transition occurs.

Mistake rate (*λ*_*M*_) is the number of mistakes that a FD makes per unit time, i.e., it represents how frequently a FD makes mistakes.

Query accuracy probability (*P*_*A*_) is the probability that the output of a FD is correct at a random time.

**Fig 2:**
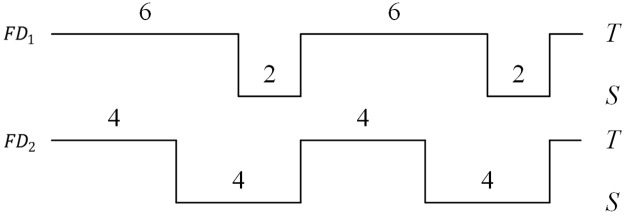
Query accuracy probability and mistake rate.

The first metric is related to a failure detector’s speed, while the remaining relate to its accuracy. In many cases, the mistake rate is not sufficient to describe the accuracy of a FD; simultaneously, the query accuracy probability is also needed. For example, Fig 2 shows that both FD_1_ and FD_2_ are detecting the node *p*. The two FDs have the same mistake rate (0.125) but different query accuracy probabilities (0.75 and 0.5).

### Adaptive failure detector

The adaptive FDs are designed to adapt to changing network conditions and application requirements [39]. In most adaptive FDs, their implementations are based on a heartbeat strategy. Existing main adaptive FDs (Chen FD, *φ* FD and ED FD) work as follows:

Chen et al. [31] proposed the QoS-based adaptive failure detection algorithm based on a probability network model. This algorithm assumes that node *p* sends heartbeat message *m* to node *q* periodically. The recent *n* heartbeat messages *m*_1_, *m*_2_, ⋯, *m*_*n*_ stored in a sliding window at *q*. *A*_1_, *A*_2_, ⋯, *A*_*n*_ are their receipt times according to *q*’s local clock. Then, the expected arrival time of next heartbeat message is estimated by:

$$EA_{\left(k+1\right)}=\frac{1}{n}\sum_{i=k-n-1}^{k}(A_{i}-\eta *i)+(k+1)\eta$$ (1)

where *η* is the sending interval, decided by the QoS requirement of user. The freshpoint *τ*_*k*+1_ of next heartbeat message is consist of *EA*_(*k*+1)_ and constant safety margin *α*. One has

$$\tau_{k+1}=EA_{\left(k+1\right)}+\alpha$$ (2)

This FD estimates the arrival time of next heartbeat message based on a constant safety margin.

In *φ*-FD, the output is a continuous value *φ* to represent the suspicion level of the monitored node, rather than a binary value simply representing true or suspect. It keeps a sliding window to save the most recent arrival time of heartbeat messages, and assumes that the heartbeat inter-arrival time follows a normal distribution. Then the value of *φ* can be calculated as follows:

$$\varphi (T_{now})=-log_{10}(P_{later}(T_{now}-T_{last}))$$ (3)

where the *T*_*last*_ is the time when the most recent heartbeat message is received, *T*_*now*_ is the current time, and *P*_*later*_(*t*) is the probability of a heartbeat message will arrive more than t time units later than the previous one. According to the assumption of heartbeat inter-arrival time, *P*_*later*_(*t*) is given by the following equation:

$$P_{later}(t)=\frac{1}{\sigma \sqrt{2\pi }}\int_{t}^{\infty }e^{-\frac{{\left(x-\mu \right)}^{2}}{2\sigma^{2}}}dx=1-F(t)$$ (4)

where *F*(*t*) is the cumulative distribution function of a normal distribution with mean *μ* and variance *σ*^2^. When the applications query the *φ*-FD at time *T*_*now*_, *φ* FD will return a value of *φ* to them. Then each application compares the value *φ* with its threshold Φ, which is given by different QoS requirements. If *φ* > Φ, a certain action is trigged. Thus, *φ*-FD can meet different QoS requirements of multiple applications simultaneously.

ED FD is similar to the *φ*-FD. The difference is in the assumption of distribution of heartbeat inter-arrival time. In ED FD, it assumes that the heartbeat inter-arrival time follows an exponential distribution. Then, the suspicion level is given by a value, called *e*_*d*_, which is calculated as follows:

$$e_{d}=F(T_{now}-T_{last})$$ (5)

$$F(t)=1-e^{-\frac{t}{\mu }}$$ (6)

where *T*_*now*_, *T*_*last*_ and *μ* have the same meaning as for the *φ* FD. For ED FD, the threshold is called *E*_*d*_.

For a FD, it will belong to the class *◇P* if it satisfies the following properties [40]:

Strong completeness: eventually every node that crashes is permanently suspected by every correct node.

Eventually strong accuracy: there is a time after which a correct node is no longer suspected by any correct node.

## Implementation

### System model

We consider a partially synchronous system consisting of a finite set of nodes ∏ = {*p*_1_, *p*_2_, ⋯, *p*_*n*_}. Each node behaves correctly until it crashes and is unable to recover. Any two nodes can be connected by an unreliable communication channel. Because most FDs are implemented using the UDP protocol, we assume that the communication channel between nodes is a fair-lossy channel [41], i.e., no message can be copied or modified and no new message can be created, and if a node *p* continues sending a message *m* to *q*, *q* will eventually receive *m*.

We assume the existence of some global time (unbeknownst to nodes), denoted as global stabilized time (GST), and that nodes always make progress; furthermore, at least *δ* > 0 time units elapse between consecutive steps (the purpose of the latter is to exclude the case where nodes require an infinite number of steps in finite time).

To simplify the description, consider a system that consists of only two nodes *p* and *q*, where *q* is monitoring *p*. Node *p* sends a message to *q* every *η* time (sending interval) or is subject to crashing. Node *q* suspects node *p* if it does not receive any heartbeat message from *p* for a period of time determined by the freshpoint.

### The implementation of 2E-FD

To improve the battery consumption of RSU with failure detector, we propose an energy-efficient failure detector 2E-FD. When a heartbeat message is sent to the receiver, the message delay *d*_*i*_ can be calculated by

$$d_{i}=T_{now}-(i-1)*\eta$$ (7)

where *η* is the sending interval, *T*_*now*_ is the arrival time of new heartbeat message. We assume that the message delay of next heartbeat message *d*_*i*+1_ is equal to *d*_*i*_, so the expected arrival time of next heartbeat message is

$$EA_{i+1}=j*\eta +d_{i}$$ (8)

According to single exponential smoothing method [42], for the each *d*_*i*_, the new predictive delay ${\hat{d}}_{i+1}$ is computed from the formula:

$${\hat{d}}_{i+1}=\alpha *{\hat{d}}_{i}+(1-\alpha )*d_{i}$$ (9)

where *α* (0 ≤ *α* ≤ 1) is a constant between 0 and 1, which controls how rapidly the ${\hat{d}}_{i+1}$ adapts to the delay change. So the safety margin (*SM*) can be estimated by:

$$SM_{i+1}=\beta \left|{\hat{d}}_{i+1}-d_{i}\right|$$ (10)

where *β* is a variable, chose such that there is an acceptably small probability that the delay for the heartbeat message will exceed timeout. At last, the freshpoint of heartbeat message (*i* + 1) can be computed by

$$\tau_{i+1}=EA_{i+1}+SM_{i+1}$$ (11)

2E-FD is unable to get the communication delay from the sender to the receiver when it is lost. To ensure the effectiveness of the proposed approach, we fill the gaps with a value computed by $d_{k}=(\frac{k-l}{2})\cdot \eta +d_{l}$, where *d*_*k*_ is the estimation of message delay of unreceived heartbeat message *m*_*k*_ from *p*, *d*_*l*_ is the message delay of receiving last heartbeat message *m*_*l*_ from *p*.

**Fig 3:**
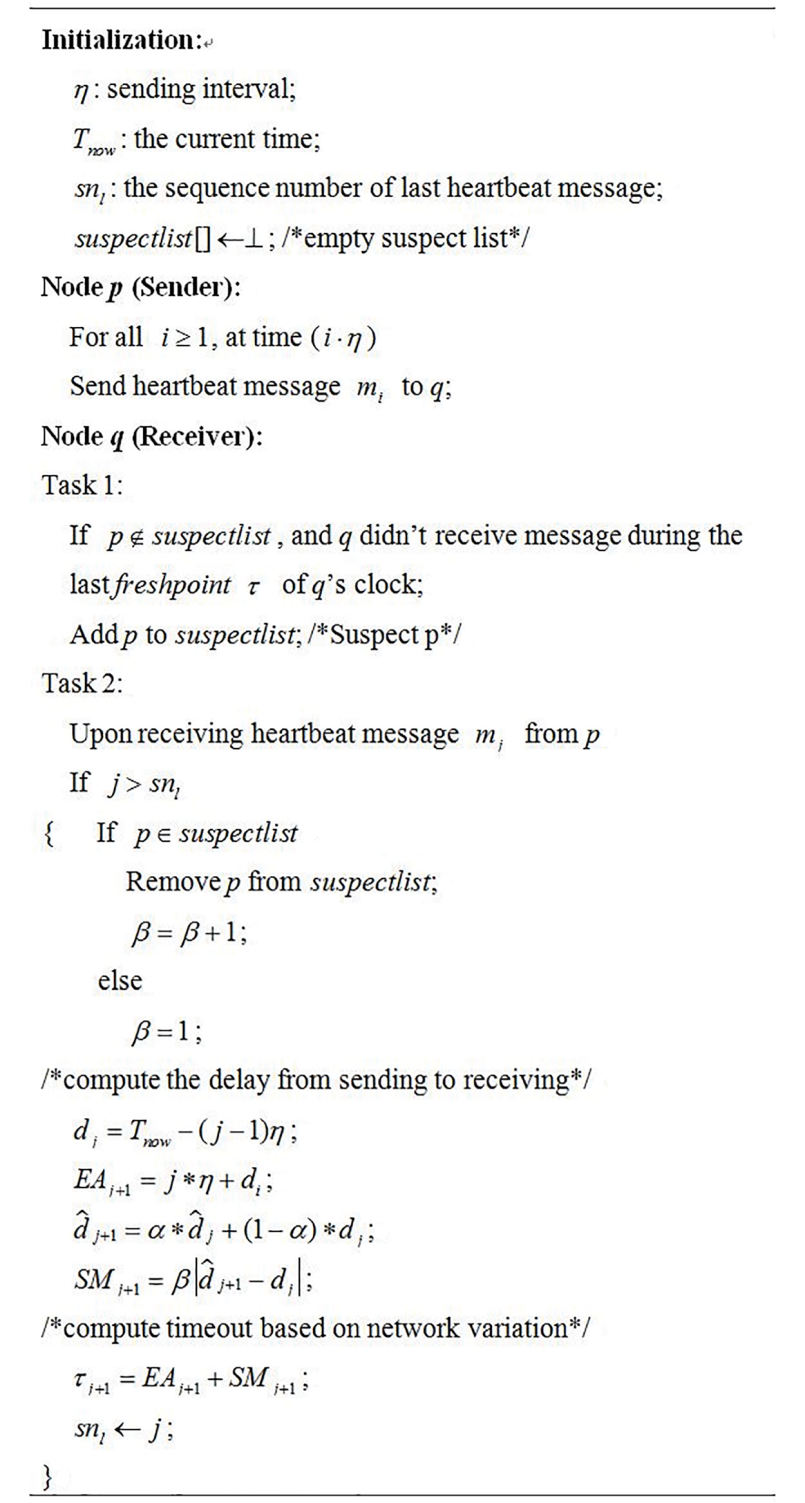
2E-FD algorithm.

2E-FD employs the heartbeat approach as the basic failure detection strategy. To simply the description, suppose the system consists of only two nodes *p* and *q*, where *q* is monitoring *p*. The detection algorithm is shown in Fig 3.

For the node *p*, it sends heartbeat message to node *q* at interval *i* * *η* (*i* ≥ 0). For the node *q*, two tasks will be executed. One task is to suspect the node *p* when *q* didn’t receive any heartbeat message during the last freshpoint *τ* of *q*’s clock. Another task is to compute the freshpoint *τ* according to the arrival time of new heartbeat message. Every time after receiving the new arrival heartbeat message, node *q* needs to compute the delay from sending to receiving and safety margin of next heartbeat message.

### Correctness proof

From the theory point-of-view, the 2E-FD can satisfy the strong completeness and eventually strong accuracy. Therefore, our FD belongs to class *◇P*, which is sufficient to solve the consensus problem. The 2E-FD implements a FD of class *◇P* under the condition of the system model defined in section 3. The evidence is as follows.

According to the system model, there is an upper bound on node speeds and on message transmission times after GST. We assume that Δ_*msg*_ is the upper bound and node *p* will not send any heartbeat message after it crashes. The heartbeat message *m*_*k*−1_ is the last heartbeat message from *p* before *p* crashes. So node *q* can calculate the freshpoint *τ*_*k*_ of next heartbeat message based on Eq (11).

$$\tau_{k}=EA_{k}+SM_{k}$$ (12)

Because *EA*_*k*_ = (*k* − 1) * *η* + *d*_*k*−1_ and *d*_*k*−1_ < Δ_*msg*_, one gets

$$EA_{k}<(k-1)*\eta +\Delta_{msg}$$ (13)

For the safety margin $SM_{k}=\beta \left|{\hat{d}}_{k}-d_{k-1}\right|$, we have

$${\hat{d}}_{k+1}=\alpha \cdot {\hat{d}}_{k}+(1-\alpha )\cdot d_{k}$$ (14)

By recursion property of equation, one gets

$$\begin{array}{ll} {\hat{d}}_{k+1} & =\alpha (\alpha {\hat{d}}_{k-1}+(1-\alpha )d_{k-1})+(1-\alpha )d_{k}\\ & =\alpha^{2}{\hat{d}}_{k-1}+\alpha (1-\alpha )d_{k-1}+(1-\alpha )d_{k}\\ & \cdots \\ & =\alpha^{k+1}{\hat{d}}_{0}+\alpha^{k}(1-\alpha )d_{0}+\alpha^{k-1}(1-\alpha )d_{1}+\ldots +(1-\alpha )d_{k} \end{array}$$ (15)

Because $d_{0}={\hat{d}}_{0}=0$,

$$\begin{array}{ll} {\hat{d}}_{k+1} & =\alpha^{k-1}(1-\alpha )d_{1}+\alpha^{k-2}(1-\alpha )d_{2}+\ldots +(1-\alpha )d_{k}\\ & =(1-\alpha )\sum_{i=1}^{k}\alpha^{k-i}d_{i} \end{array}$$ (16)

Therefore, using the same methods, we get

$${\hat{d}}_{k}=(1-\alpha )\sum_{i=1}^{k-1}\alpha^{k-i}d_{{}_{i}}$$ (17)

Because 0 ≤ *d*_*i*_ < Δ_*msg*_, therefore

$$0\leq {\hat{d}}_{k}<\alpha (1-\alpha^{k-1})\Delta_{msg}$$ (18)

From inequalities (18), we get

$$SM_{k}<\beta \left|\alpha (1-\alpha^{k-1})\Delta_{msg}\right|=\beta \cdot \alpha (1-\alpha^{k-1})\Delta_{msg}$$ (19)

Thus, the freshpoint *τ*_*k*_ of next heartbeat message is

$$\tau_{k}=EA_{k}+SM_{k}<(k-1)\cdot \eta +\Delta_{msg}+\beta \cdot \alpha (1-\alpha )\Delta_{msg}$$ (20)

From inequation (20), all components of *τ*_*k*_ are bounded, so we can deduce *τ*_*k*_ is bounded. If for each message *m*_*k*−1_ received from node *p*, node *q* activates a bounded timeout, then there is a time after which *q* suspects *p*, if it receives no new message from *p*. Thus the 2E-FD satisfies the strong completeness property.

Every time *q* time out and *p* is correct, then *β* is increased. There is a time *t*_*bound*_ where safety margin *SM* is larger than Δ_*msg*_. The heartbeat message from node *p* must be received by node *q*. As a result, node *q* can avoid false detection, and *SM* stops increasing. Thus, when *SM* becomes higher than Δ_*msg*_, a correct node is no longer suspected by any correct node. This indicates the 2E-FD satisfies the eventually strong accuracy property.

## Evaluation and performance

In this section, we will evaluate and analyze the performance of 2E-FD through comparative experiments. First, the 2E-FD was compared with the failure detection algorithms in terms of detection time, mistake rate and query accuracy probability. Then, we tested the battery consumption of RSU with different failure detection algorithms in a simulation environment.

### 2E-FD performance evaluation and comparison

To enhance the authenticity of the experiment, we referred to the method in paper [43] that used the same trace files to replay different schemes of FDs and calculated the QoS metrics. This method ensures that all schemes of FDs are compared in the same network condition. The trace files are obtained from two network conditions (Wireless and WAN). For the accrual FDs (*φ*-FD and ED FD), it is necessary to compute the parameters of FD based on multiple history messages. In these experiments, *φ*-FD and ED FD shared the same fixed window size (1000), while the Chen FD used two different sliding windows (*WS* = 1 and *WS* = 1000). Furthermore, we did not analyze the sampled data until the sliding window was full, since the behavior of the FDs is stable only after that moment. The parameters of FDs are configured as follows: the basic parameters of 2E-FD are set as *α* = 0.85, and in order to find the best QoS and compare with the others, here *β* ∈ [10^−6^, 10^6^]. For *φ*-FD, the parameters are set the same as in [32]: Φ ∈ [0.5, 16]. For Chen FD, the parameters are set the same as in [31]: *α* ∈ [0, 10000]. For ED FD, the parameters are set the same as in [33]: *E*_*d*_ ∈ [10^−4^, 10].

For the Wireless case, two nodes are selected to represent the detecting node and monitored node respectively. The two nodes communicated through a WiFi (802.11g) network. The detecting node was equipped with a 900MHz ARM Cortex A7 processor, 1G RAM and Cent OS 6.5 operating system. While the monitored node was equipped with a 2GHz Intel Xeon processor, 1G RAM and Cent OS 6.5 operating system. The heartbeats were sent with a target of one heartbeat every 100.5ms (standard deviation: 7.87ms; min.: 0.002ms; max.: 948.96ms). During the experiment, the round-trip time (RTT) was measured to be at a low rate. The average RTT was 1.83ms, with a minimum of 1.175ms, and a maximum of 21.953ms. More than 1 million heartbeats were sent.

**Fig 4:**
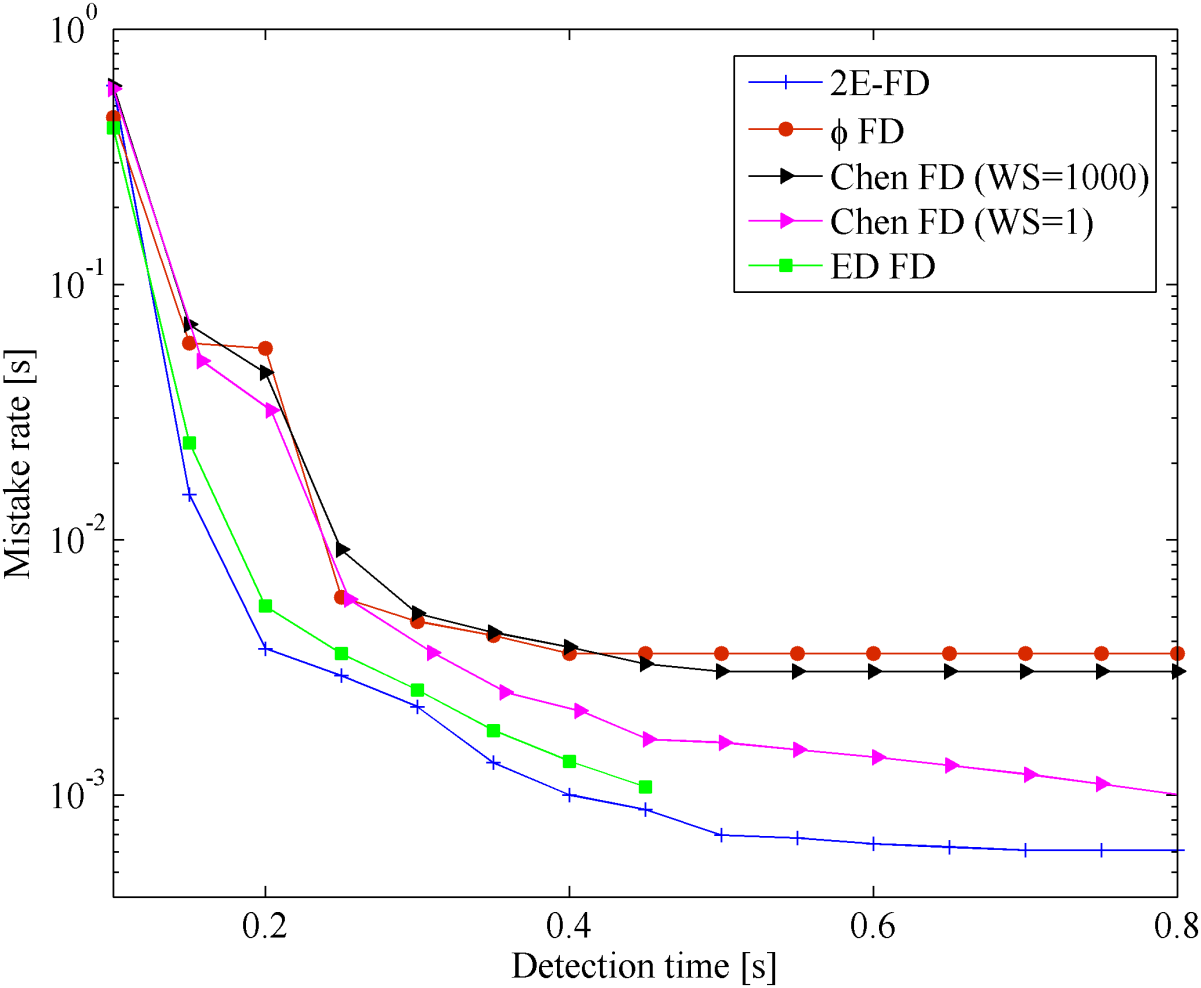
Mistake rate vs. detection time in Wireless.

**Fig 5:**
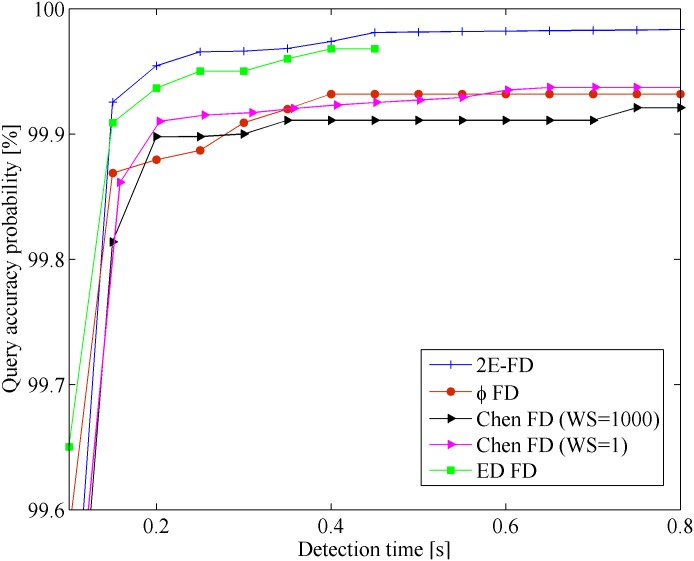
Query accuracy probability vs. detection time in Wireless.

Fig 4 shows the results of the mistake *λ*_*m*_ vs. detection time *T*_*D*_ in the Wireless scenario. The x-coordinate represents the detection time, and y-coordinate represents the mistake rate. Fig 5 shows the results of query accuracy probability *P*_*A*_ vs. detection time *T*_*D*_ in the same scenario.

From the Figures, we found that all the FDs follow the same general tendency. However, our FD outperforms the others in the Wireless scenario. This improvement is because most late heartbeats were caught by the freshpoint under the same network conditions. In Fig 4, when *T*_*D*_ < 0.2*s*, the mistake rate of 2E-FD has obvious decrease than other FDs. This is because that our FD can quickly adapt to the network conditions than other FDs. From Fig 5, our FD has higher query accuracy probability than others. When 0.15*s* < *T*_*D*_ < 0.4*s*, our FD and ED-FD seem to have similar query accuracy probability. At the same time, it is clear that the performance of Chen FD with *WS* = 1 is better than Chen FD with *WS* = 1000 in terms of mistake rate and query accuracy probability. This may be because that many burst data and too old data affect the calculation of freshpoint when sliding window size increases.

For the WAN case, the experiment was carried out on two nodes respectively located in Japan and Switzerland [32]. In the experiment, all heartbeat messages were transmitted using UDP protocol, and the monitored node *p* sent heartbeat messages to node *q* at the interval of 100ms, and *q* recorded all the arriving time of the received messages into the trace file according to its local clock. The experiment lasted a week, and more than 5 million heartbeat messages has been collected, where the average RTT is 283.3ms and message loss rate is approximately 0.4%.

**Fig 6:**
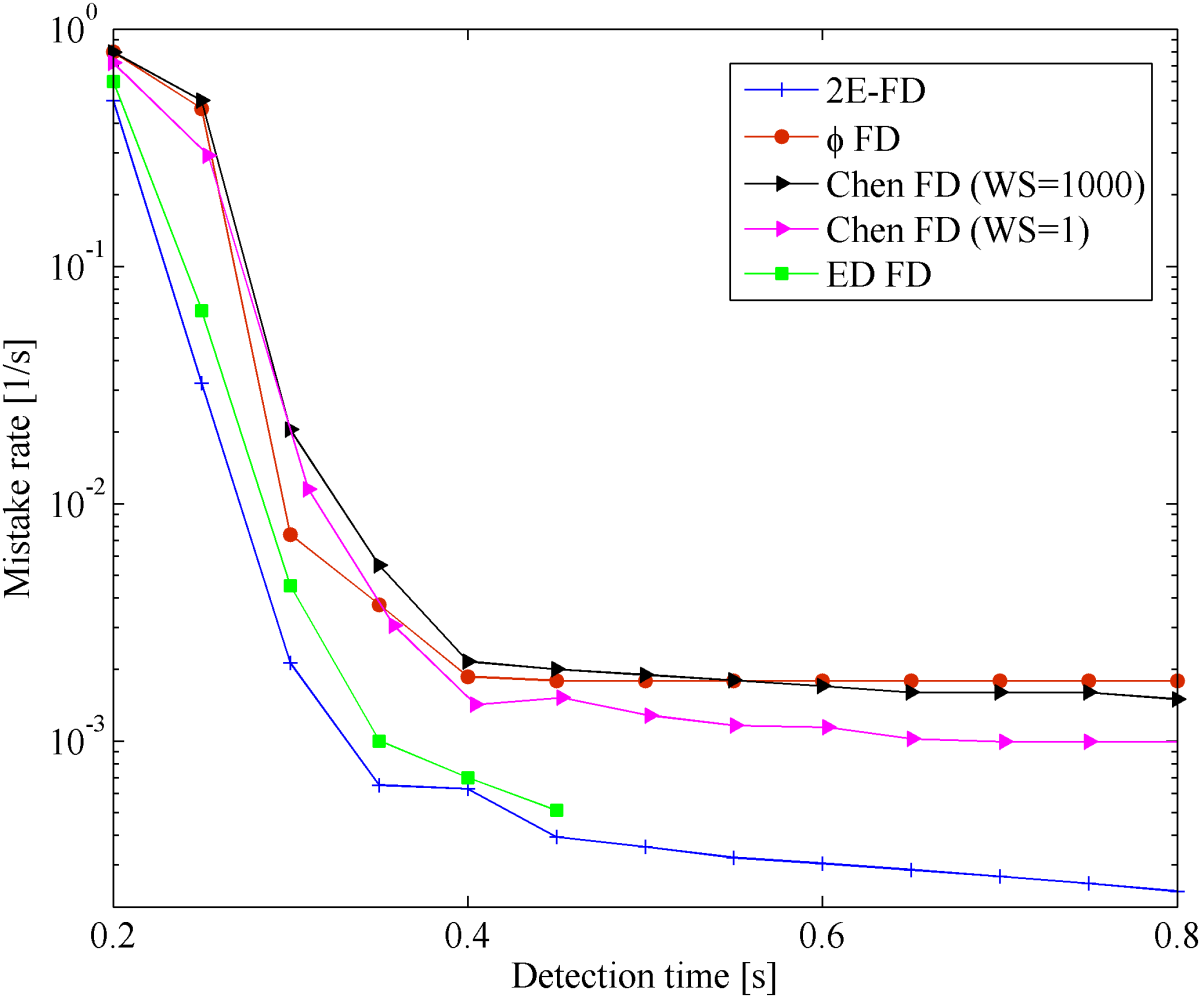
Mistake rate vs. detection time in WAN.

**Fig 7:**
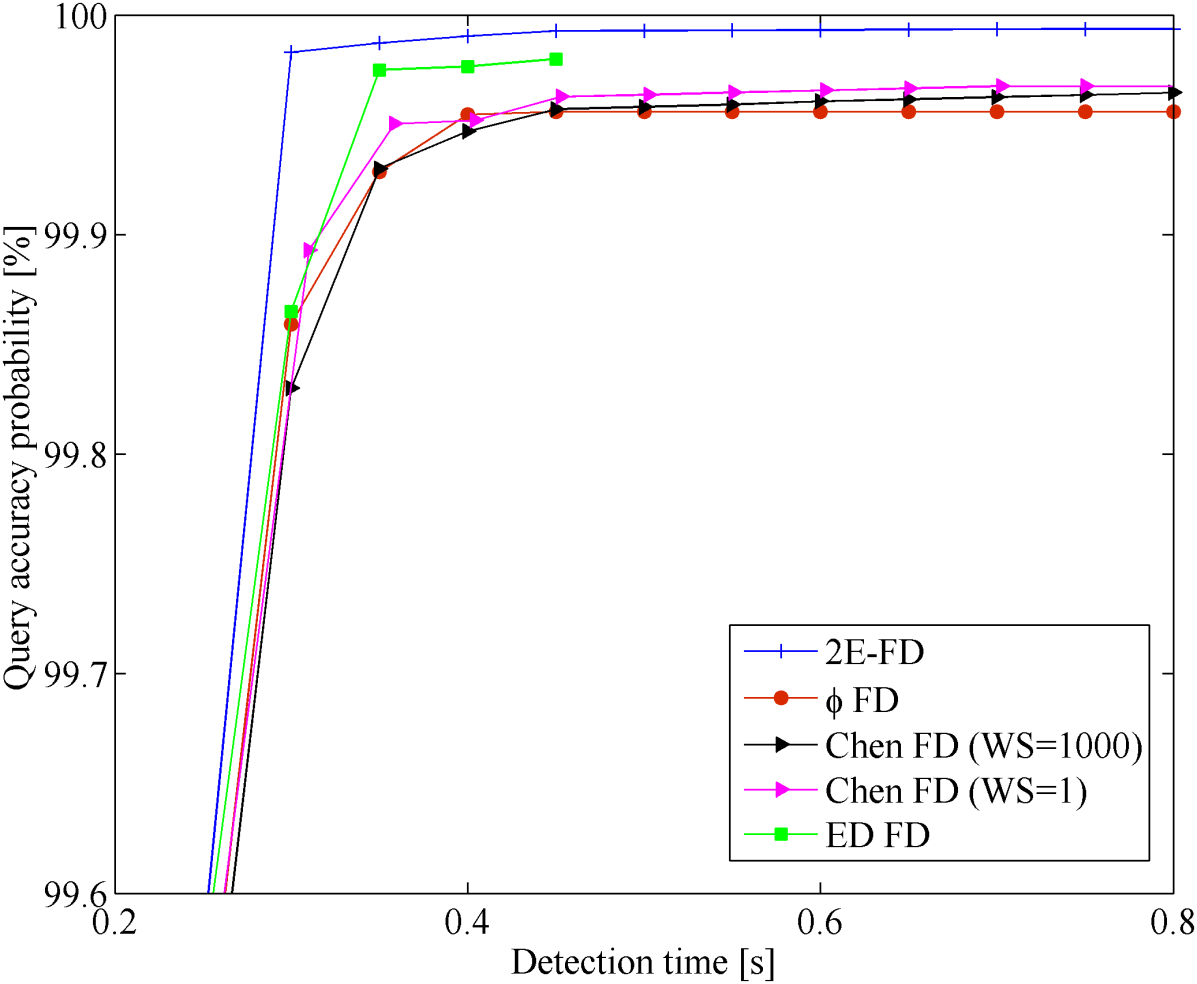
Query accuracy probability vs. detection time in WAN.

Figs 6 and 7 show the results of the mistake rate *λ*_*m*_ and query accuracy probability *P*_*A*_ vs. detection time *T*_*D*_ in the WAN scenario. Similar to the result in the Wireless scenario, the mistake rate and query accuracy probability of all of the FDs have an identical tendency with increasing detection time. In Fig 6, the 2E-FD presents the lowest mistake rate (an improvement of up to 50%). It shows that our predictive method can improve the detection accuracy effectively. In Fig 7, our FD has an obvious improvement compared with ED FD when *T*_*D*_ < 0.35*s*. Furthermore, when the mistake rate or query accuracy probability is the same, our FD performs shorter detection time. In the WAN, 2E-FD behaves better than the others FDs in terms of quick speed and high accuracy.

### Comparison of battery consumption

RSUs are static devices that disseminate the data stored in the infrastructure to the passing-by vehicles periodically [28]. In addition, RSU may exploit Infrastructure-to-Vehicular single-hop IEEE 802.11-like wireless links for data dissemination [11]. Based on these, we simulated the working environment of RSU by organizing several nodes. These nodes were connected by wireless link. One node was selected to represent the RSU, and the other nodes represented the vehicles. The RSU, which was equipped with 500mAh battery, was responsible for disseminating data to vehicles [25,28]. The vehicles may be failure via the fault injection method. At each experiment, we deployed different FD to the RSU and measured the running time of RSU.

**Fig 8:**
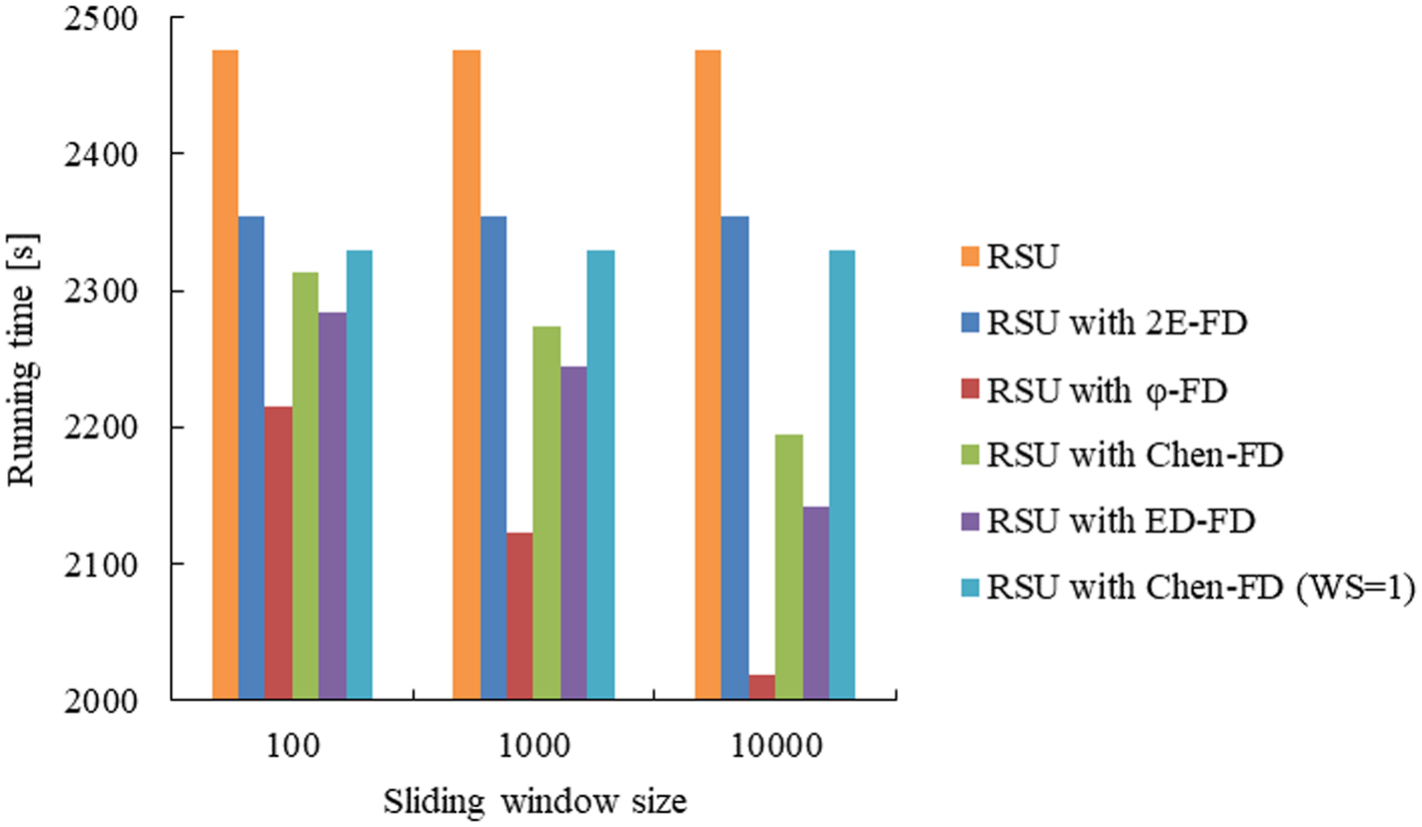
Running time of RSU vs. sliding window size for different FDs.

For the accrual FDs (*φ*-FD and ED FD) and Chen FD, they need to calculate the detector parameters and maintain the history window of detection messages at every detection period. We have selected different sliding window size setting (from *WS* = 100 to *WS* = 10000) and have made a detailed comparison of battery consumption of RSU. To make the experiment general, we re-did the experiments 5 times with the same environment, and the same parameters for each failure detection algorithms. Finally, we recorded the running time of RSU with different failure detection algorithms (as shown in Fig 8).

From the Fig 8, it shows that the running time of RSU without any FD is the longest and improved by 6% compared to the RSU with 2E-FD. For the RSU with FD, the running time of RSU with *φ*-FD is the shortest, and it quickly decreases as the sliding window size changes. This is because that the battery consumption of RSU is exacerbated when the parameters of normal distribution model are calculated. The calculation of parameters needs the statistical data from the entire window. While the running time of RSU with 2E-FD is the longest (an improvement of up to 12% than *φ*-FD when the sliding window size is 10000), and it is not affected by sliding window size. For the RSU with Chen FD (*WS* = 1), its running time is not affect by sliding window size too. However, its running time is shorter than the RSU with 2E-FD. This might be because that the accuracy of 2E-FD is higher than Chen FD (*WS* = 1) due to the dynamic safety margin. 2E-FD avoids sending more data to the failure nodes so as to save the battery consumption of RSU. The RSU in the experiment shown in Fig 8 at most connects 10 nodes. In vehicular cloud computing, in order to maintain the connectivity of systems, each RSU is required to connect many vehicles. Therefore, the fact that 2E-FD can reduce battery consumption of RSU is more significant in real systems.

## Conclusion

Failure detection plays a very important role in vehicular cloud computing. In this paper, we introduced the energy-efficient failure detector (2E-FD). It has been proven that 2E-FD is a failure detector of class *◇P*. By using the prediction of last message and the dynamic safety margin, the 2E-FD can quickly adapt to the network conditions and provide acceptable QoS failure detection service. Moreover, 2E-FD is able to save the battery consumption of RSU because it does not need to compute the distribution parameters and maintain the sliding window. Through the comparative experiments, the results showed that 2E-FD demonstrates a better performance in terms of detection speed, accuracy and battery consumption. Therefore, 2E-FD is suitable to be layout in the vehicular cloud computing for providing failure detection service.

## Supporting information

S1 File Wireless. (ZIP)
